# Far from reality, or somehow accurate? Social beliefs and perceptions about traffic crashes in the Dominican Republic

**DOI:** 10.1371/journal.pone.0282601

**Published:** 2023-04-07

**Authors:** Francisco Alonso, Mireia Faus, Sergio A. Useche

**Affiliations:** 1 INTRAS (Research Institute on Traffic and Road Safety), University of Valencia, Valencia, Spain; 2 Faculty of Psychology. University of Valencia, Valencia, Spain; Al Mansour University College-Baghdad-Iraq, IRAQ

## Abstract

**Background:**

Despite the considerable effort made during the last decades, emerging countries are still among the highest road safety concerns because they still account for most of the deaths caused by traffic crashes. Various studies suggest that one of the factors involved in this negative outcome could be road safety. However, this issue remains pending to be addressed in most emerging countries, including the Dominican Republic.

**Aim:**

This study aimed to assess the beliefs and perceptions of Dominicans regarding some key road risky-related issues and to discuss them in the light of objective data.

**Methods:**

For this cross-sectional study, the responses by a full sample of 1,260 Dominicans (50.1% men, 49.9% women) with a mean age of 39.4 years participating in a set of surveys conducted across the country, were used.

**Results:**

Although Dominicans (especially women) seem to attribute high importance to road crashes, there is a low perceived likelihood of getting involved in a traffic crash. As for subjective versus objective data comparisons, perceived crash features and objective crash report data considerably match. However, the numbers largely differ in terms of crash frequency and importance and relevance given to road crashes, and their consequences. Further, perceptions of traffic violations and lack of law enforcement were pertinent predictors of the degree of relevance attributed to traffic crashes.

**Conclusions:**

Overall, the results of this study suggest that, despite a relative awareness of their actual traffic crash features, Dominicans systematically underestimate the causes, frequency, and consequences of these crashes, including yearly fatality rates. These outcomes suggest the need to strengthen road safety awareness and beliefs in further road safety actions and policymaking in the region.

## Introduction

One of the highly prevalent and most concerning safety-related challenges of Low and Middle-Income Countries or LMICs (AKA emerging countries) is, nowadays, their overrepresentation in terms of traffic crashes and fatalities [1]. In fact, while road safety numbers keep slightly improving in high-income economies, 90% of deaths due to traffic crashes globally remain concentrated in countries in LMICS [2]. This figure becomes particularly relevant if it is considered that emerging countries count, altogether, for one-third of the automotive fleet worldwide [3].

These facts suggest that, like other spheres (including employment, poverty and access to goods and services), transport is one of the contexts in which great inequalities are deepened, especially since emerging countries consistently show certain characteristics that make them particularly vulnerable [4]. One of them is that due to an increase in the motorization over last few years, policymaking, enforcement, and social awareness tend to get left behind in these countries [5]. Moreover, multiple pieces of evidence illustrate that existing economic limitations in these regions do not allow for maintaining road infrastructures in ideal conditions [6], explaining a highly vulnerable, unstable, and ‘volatile’ state of affairs in terms of road safety [7, 8]. For instance, in 2015, the mortality rate in low and medium-income regions was 34 per 100,000 inhabitants. Meanwhile, in high-income countries, the numbers were as low as 8 per 100,000 inhabitants [9].

The World Bank (2020) claims that incomes from developing countries could increase substantially if the number of road crashes were reduced [10, 11], but critical constraints, such as the ‘endless loop’ still threaten this process: as developing regions do not have enough resources to establish effective preventative measures, it is harder to reduce their number of road crashes, which in turn tend to impoverish them further [12, 13]. Indeed, a recent report states that the potential growth of the gross domestic product (GPD) per capita of the countries which do not invest in road safety could even be reduced by between 7% and 22% [10]. In this regard, recent studies suggest that investments in crash prevention should be a critical objective for government authorities from emerging countries [14–17].

### Importance of risk perception in the change of attitudes and behaviors

One of the core features of road risk perception is the adjustment between the subjective perception of hazards in several situations, practices and scenarios and the actual threat they represent to road users. Said otherwise, the more realistic the risk-related perceptions, the more positive the users’ behavior would be expected to be [18–20]. Therefore, some studies have emphasized the importance of making citizens more aware of certain social and health-related issues, usually focusing on road crashes [21]. Thus, identifying possible dangers could be considered a suitable first step for individuals to develop safety-related attitudes and behaviors [22–25].

Nevertheless, on many occasions, the risk perception of subjects over safety and health-related issues might be distorted by cognitive bias [26]. A clear example of this, is the widespread underestimation of cardiovascular problems. While cardiovascular diseases represent one of the leading causes of death worldwide, individuals tend to rank other matters (e.g., cancer, AIDS, terrorism, or delinquency) as of greater importance, frequently contributing to inhibiting their care-related behaviors and preventive actions [27–30].

Specifically, in the field of road safety, empirical evidence supports that a low-risk perception of crash likelihood is, in most cases, related to (e.g.) performing aggressive and dangerous behaviors on a more regular basis, as well as to lower overall law compliance while driving, cycling or walking [31, 32]. Further, risk perception has been addressed and supported as a critical factor for practically all groups of road users, since there have been investigations with similar results on pedestrians [33, 34], cyclists [35, 36], private car drivers [37], motorcyclists [38] and professional drivers [39, 40].

### Approaching the Dominican case in figures

Whereas international applied research on risk perception in traffic has been undertaken in high-income countries, the pieces of evidence from LMICs remain considerably scarce and disproportionate to the social problems affecting them, thus inviting researchers to fill scientific gaps such as the lack of evidence on transportation, human behavior, and road safety [41–43]. However, some data (most of them retrievable from official reports and public repositories) might help illustrate the state of affairs in transport and road safety of the country.

In the Dominican Republic, the road crash-related mortality rate is currently 29.3 deceased for each 100,000 [44]. It means the highest number in the American continent and one of the highest globally [10]. The transport dynamics, the estate of the vehicles and the conditions of the infrastructures are some of the causes that are influencing these negative trends [6].

The country has diverse public transport systems such as motorcycle taxis, public buses and metro in bigger urban areas [45]. Nevertheless, the poor conditions of vehicles, security issues, and low accessibility of public transport frequently result in users opting for private vehicles [46]. This also explains a growing trend in the country’s vehicle fleet during the last decade. Nowadays, more than 60% of the population has their own vehicle, although the figure is ’misleading’ since they are mostly low-cost motorcycles—with low mechanical standards and safety features [47]. This is, however, something not unique to the country but rather a widespread phenomenon observable in a great part of Latin American LMICs, which share many characteristics, patterns, and problems in the transport domain [48–50]. These dynamics also make these countries very likely to report multiple mobility-, road safety-, and pollution-related issues [51, 52].

### Study aim

The core aim of this study was to assess the beliefs and perceptions of Dominicans regarding some key road risky-related issues and to discuss them in the light of objective data.

## Methods and materials

### Participants

The sample used for this study consisted of 1,260 adult inhabitants of the Dominican Republic with a mean age of *M* = 39.4 (*SD* = 15.4) years. 49.9% of them were women, and 50.1% were men. A more detailed set of basic demographics of our participants is presented in Table 1. It is worth mentioning that the sample had a proportional distribution to the population in terms of sex, age (adults), province, and habitat, using the data provided by the last national census (ONE).

**Table 1:** Sociodemographic data of the study sample.

Factor		N	%
**Sex**	Men	631	50.1%
	Women	629	49.9%
**Age range**	18 to 24 years old	261	20.7%
	25 to 34 years old	318	25.2%
	35 to 49 years old	367	29.1%
	50 to 64 years old	211	16.7%
	More than 65 years old	103	8.2%
**Habitat**	Urban	996	79%
	Rural	264	21%
**Licensed driver?**	Yes	289	22.9%
	No	971	77.1%
**Frequent driver?** (More than once a week)	Yes	441	35%
	No	819	65%

Nationwide representativeness was achieved by setting the minimum sample size needed to be about *n* = 680 individuals, assuming a level of confidence of 99%, a maximum margin of error of 5% (α = .05), and a beta (β) of .20, which allows for 80% power. Participation was voluntary and anonymous. The management of personal information was carried out in compliance with the existing laws on data protection and ethical guidelines.

### Design, procedure, and instruments

**Fig 1:**
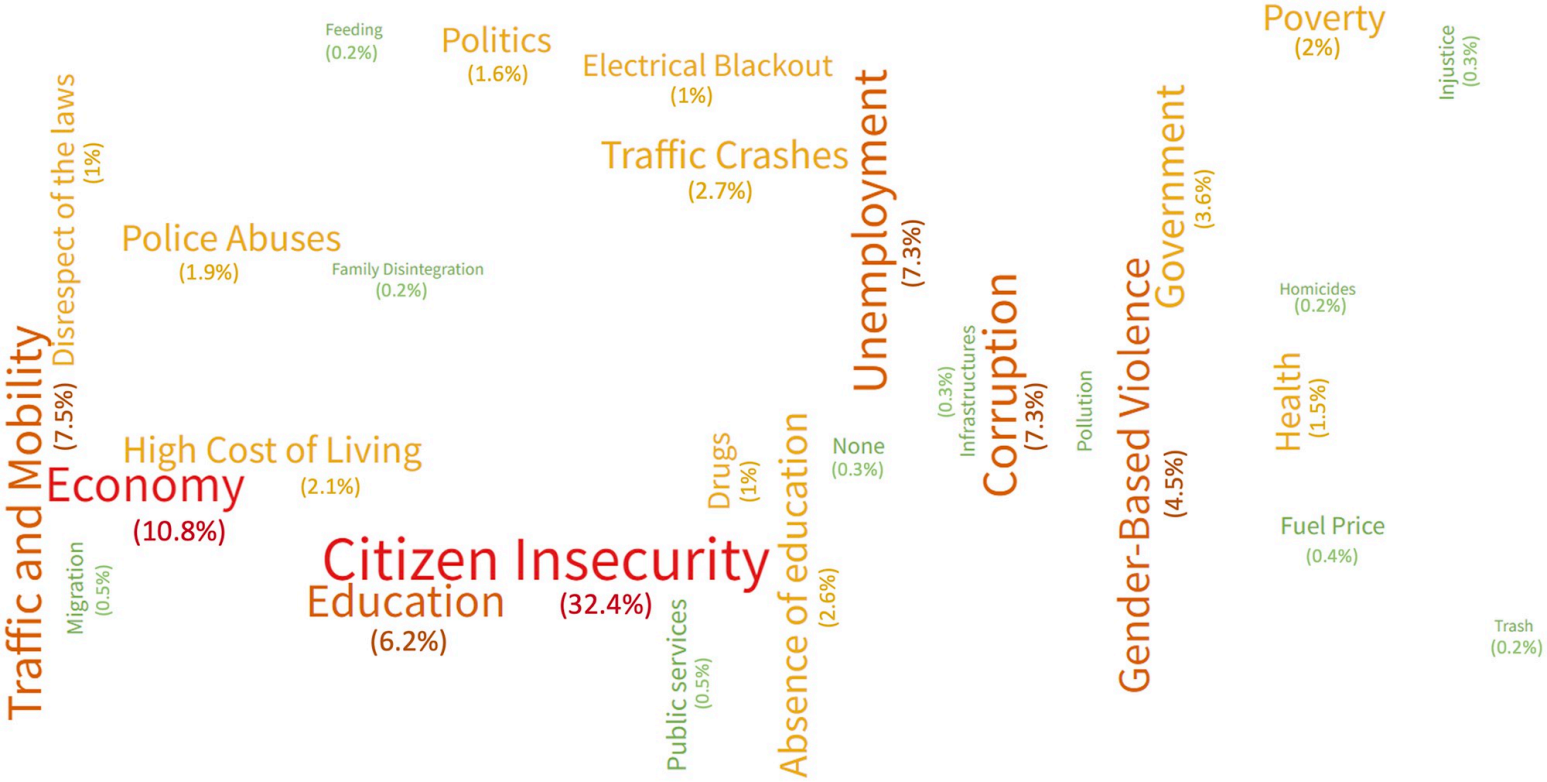
Main social and health-related current concerns, as reported by Dominican citizens. Bigger fonts/words represent the most frequently quoted matters.

The data was collected using a CAPI (Computer Assisted Personal Interviewing) system. Each application of the survey was systematically recorded and geo-referenced so that system recording issues and missing data were kept to a minimum. The survey was composed of 25 items to assess Dominican citizens’ perceptions of the characteristics and prevalence of road accidents in the country. The survey was administered in September to November 2020, and the average time required by participants to complete the questionnaire was 8 minutes. To test the reliability and validity of the questionnaire, a pre-test was carried out with a small sample (n = 42 participants) to detect possible difficulties in the comprehension of the items, as well as other potential problems with the survey. After this pre-test, the questionnaire was finalised, which specifically consisted of the following sections:

- *Sociodemographic variables and driving data*: sex, age group, habitat, driving licensing, driving frequency. These questions had closed response options for participants to select the option that matched their personal characteristics.
- *Perceptions on the awareness/importance attributed to road crashes*: the following were used: (*i*) open questions on the social and economic issues that were considered to be the most relevant in the country; and (*ii*) structured questions on the importance given to traffic crashes using a scale of Likert from 0 to 10. Participants were asked to point out specific issues that were more important than crashes through open questions (please refer to Fig 1 for the full list).
- *Beliefs on the prevalence of road crashes*: participants were asked to estimate/guess the approximate number of people who died in the Dominican Republic because of road crashes the year before the study. These items are presented in the form of open-ended questions for participants to state the figure they believe fits the objective data on this issue.
- *Beliefs on traffic crash characteristics*: participants’ statements on the perceived features of crashes most commonly occurring in the country (*what does a ‘standard crash” look like*?). Specifically, questions about their core features addressed: the most frequently involved types of vehicles and road users, types of road, temporal-spatial features (day, time of the day), reason(s) for such journeys, dangerous maneuvers (risky behaviors) perceived as the most frequently performed by road users, crash responsibility and their most relevant consequences. These items were presented as closed-ended questions.
- *Crash risk-enhancing factors*: Finally, participants were encouraged to assess their perceived impact of five literature-based crash contributors on the actual crashes occurring in the country: driving violations, pedestrian violations, vehicle issues, infrastructure issues, and lack of law enforcement.

### Data processing

After careful data curation, in which only a few missing data and recording inconsistencies were found, a descriptive analysis of the questionnaire variables was carried out. Namely, the data corresponding to the perceived importance of traffic crashes, their relationship with other problems in the country, as well as the attributions on their most frequent features and scenarios, were calculated by means of basic procedures (i.e., means, frequencies/percentages, and plots).

Once the basic statistical parameters were checked and/or statistical corrections (i.e., logarithmic transformation of non-normally distributed variables) were performed, ANOVA tests were used to compare mean values of continuous variables in accordance with categorical factors, such as sex, licensing status, and driving frequency. Finally, a multivariate linear regression (MLR) modeling procedure was carried out to assess the effect of several road safety-related perceptions over the importance attributed to traffic crashes. Statistical analyses were performed using © IBM SPSS (Statistical Package for Social Sciences), version 28.0.

### Ethics

The research protocol used for this study was reviewed by the Ethics Committee of the Research Institute on Traffic and Road Safety at the University of Valencia, guaranteeing its compliance with the Declaration of Helsinki, and its adherence to general ethical norms applying to this type of (cross-sectional and questionnaire-based) research (IRB approval number: HE0001251019). All participants granted and signed their written informed consent during the data collection phase.

## Results

The descriptive outcomes of this study show that, although the overall Dominican population self-reports relatively high importance to traffic crashes as a social concern (*M* = 7.96; *SD* = 3.03), there are significant differences in the valuations given between men and women (F_(1,1259)_ = 7.665; *p* = 0.06). In these sex-based differences, women tended to uniformly report higher scores (*M* = 8.39; *SD* = 2.70) than their men counterparts (*M* = 7.69; *SD* = 3.12). There were also differences according to age (F_(4,1259)_ = 7.154; *p* < .001). Younger age groups are more concerned about this issue than older age groups. Specifically, young people aged 18–24 years (*M* = 8.40; *SD* = 2.50) and the group aged 25–34 years (*M* = 8.39; *SD* = 2.70), have significantly higher scores than adults aged 50–64 years (*M* = 7.54; *SD* = 3.41), and people over 65 years (*M* = 6.96; *SD* = 3.61).

Secondly, the relevance given to crashes was compared with other issues of interest that also affect public health and safety in the country, such as insecurity, poverty, unemployment, and various diseases, among others. Thus, traffic crashes proved to be the most relevant issue for only 2.5% of the participants, as shown in Fig 1. Specifically, citizen insecurity and delinquency particularly stand out (29.7%) along with the current economic situation (9.8%). It is also worth noting that 6.9% of participants mentioned aspects related to road traffic and mobility.

The results slightly vary when asking specifically about issues they consider more important than traffic crashes. The most relevant ones are delinquency (17.5%) and gender violence (17.5%). Meanwhile, 13.3% of participants rank them first, i.e., considering there is no bigger issue in the country than road crashes.

### Road safety-related beliefs: Comparing citizens’ perceptions with official data

This descriptive analysis aimed at assessing the coherence between the degree of concern raised by traffic crashes and the actual objective data of the country regarding various public health or risk-related issues affecting citizens’ health and welfare. Overall, traffic crashes were ranked as the eighth problem in terms of importance, as seven other categories (i.e., delinquency/citizen insecurity, drug trafficking, corruption/fraud, life cost, domestic violence, homicide/suicide, and education shortcomings) were listed before.

This way, and particularly speaking about non-natural death causes, traffic crashes would occupy a considerably residual place, even though objective data shows they remained the main contributor to the issue during the seven last years before conducting the study, with an average of 1,812 deaths per year during the last decade. Table 2 summarizes the main causes of non-natural deaths registered in the place of the crash in the Dominican Republic.

**Table 2:** Number of deaths caused by an external source registered in the place of the crash (objective data).

Death cause	2011	2012	2013	2014	2015	2016	2017	2018	2019	2020
Traffic crashes	1,833	1,768	1,892	1,855	1,946	1,991	1,585	1,418	2,100	1,732
Homicide	2,517	2,268	1,990	1,810	1,680	1,616	1,561	1,390	1,232	1,136
Suicides	637	638	567	557	547	569	575	648	607	597
Drownings	349	338	330	289	293	324	305	251	266	276
Electrocutions	193	207	216	175	160	170	158	189	175	173

*Notes*: Data from the National Statistical Office (2021) [53].

There are diverse beliefs about the prevalence of road crashes in the Dominican Republic. A large proportion of respondents underestimate the number of annual fatalities resulting from road crashes. 78.1% believe there were fewer than 2000 deaths due to this cause in the year prior to survey administration. Particularly noteworthy is the 26.8% of participants who think this data was below 100 deaths.

**Fig 2:**
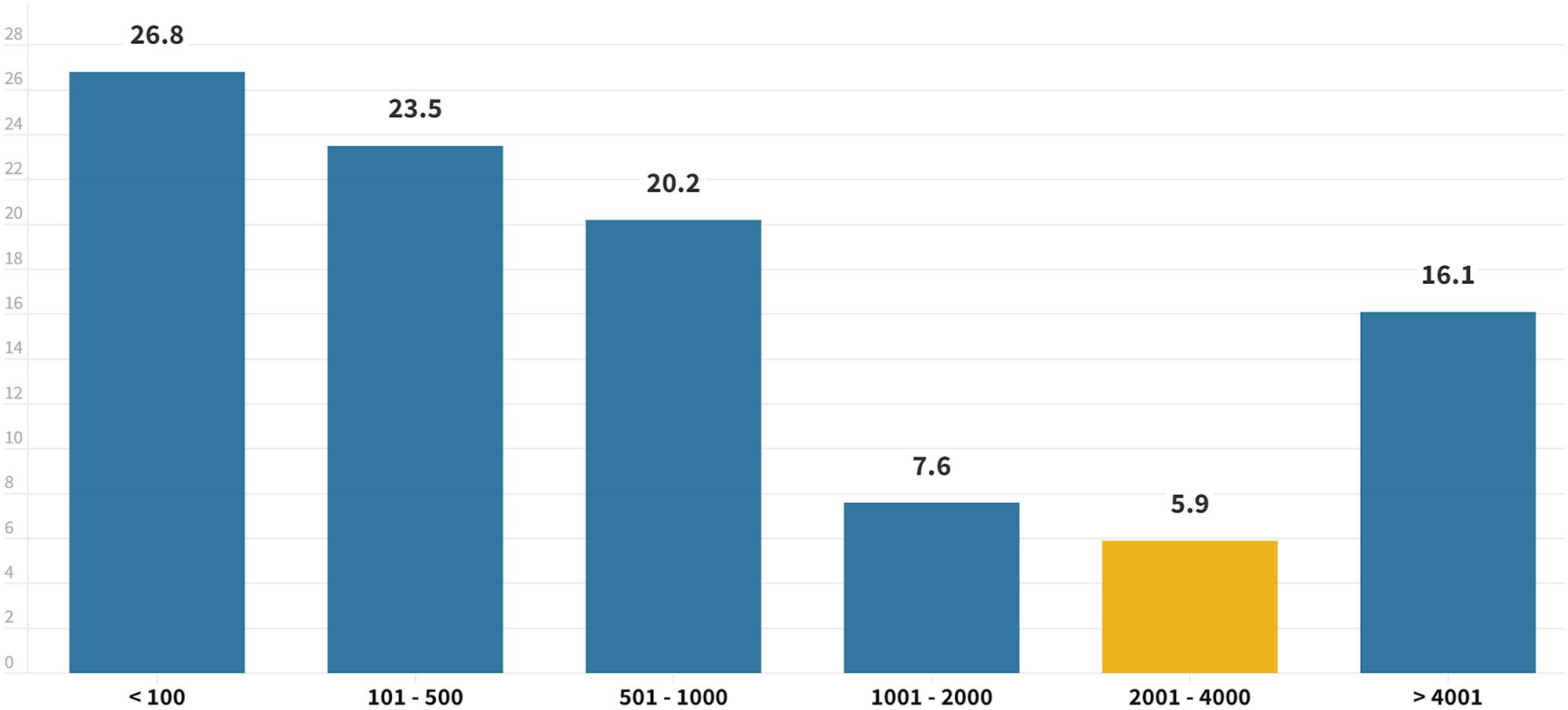
Number of yearly road fatalities estimated by study participants. The most common interval was 0–100, while it corresponds to 2001–4000 deaths.

These results contrast substantially with the real data. In fact, only 5.9% of those surveyed had a perception in line with reality since they stated data of between 2,000 and 4,000 deaths per year. There was also a part of the sample that overestimated this question, responding with values higher than the actual ones (16.4%) (Fig 2). There were no significant differences in sex, habitat, driving licensing status, and usual driving.

Afterward, questions were set out to get to know the subjective perceptions of Dominicans regarding the characteristics and typology of road crashes that happened in their country (Table 3). Practically, the sample considers that young people between 15 and 25 years old are the ones most involved in crashes (88.3%). Motorcycles are the vehicle regarded as the more significant cause of traffic crashes, no matter if they are driven by private drivers (69.6%) or professional ones (21.3%). More crashes are perceived on the road than in urban areas, particularly on highways (56.1%) and primary roads (30.5%). Lastly, participants considered holidays (76.5%) and night hours (54.3%) to be more dangerous and prone to register road crashes.

**Table 3:** Typical crash-related beliefs (hypothesized crash configurations) in the Dominican Republic.

Crash features commonly attributed by Dominicans*		Frequency	Percentage
Age Group (Common victims)	Children (to 14 years old)	39	3.1%
	Young people (between 15 to 25 years old	1112	88.3%
	Adults between 26 to 40 years old	84	6.7%
	Adults between 41 and 60 years old	10	0.8%
	Aging adults (> 60 years old)	15	1.2%
Type of vehicle (Whose users commonly constitute crash victims)	Bus	32	2.5%
	Van	9	0.7%
	Public car	17	1.3%
	Private car	53	4.2%
	Motorcycle taxi	269	21.3%
	Private motorcycle	877	69.6%
	Bicycle	3	0.2%
Zone	Urban	463	36.7%
	Rural	797	63.3%
Type of road	Highways or freeways	447	56.1%
	Ring roads	34	4.3%
	Main roads	243	30.5%
	Local roads	49	6.1%
	Country lanes	24	3.0%
What days?	Working Day	296	23.5%
	Holidays	964	76.5%
When?	Day	576	45.7%
	Night	684	54.3%

Notes:

*Participants were asked to report the crash feature they believed were the most frequently seen (only one a category) in traffic accidents occurring in the Dominican Republic.

In addition to the hypothesized data provided in Table 3, the types of displacement (trip reasons) perceived as the riskiest were those related to leisure purposes (25.8%), followed by work/commuting journeys (19%) and long-haul touristic trips (8.5%). In regard to risk manoeuvres potentially causing crashes, perceptions were varied. Risky overtaking (35.2%) and reckless motorcycle riding (22.4%) stand out.

**Fig 3:**
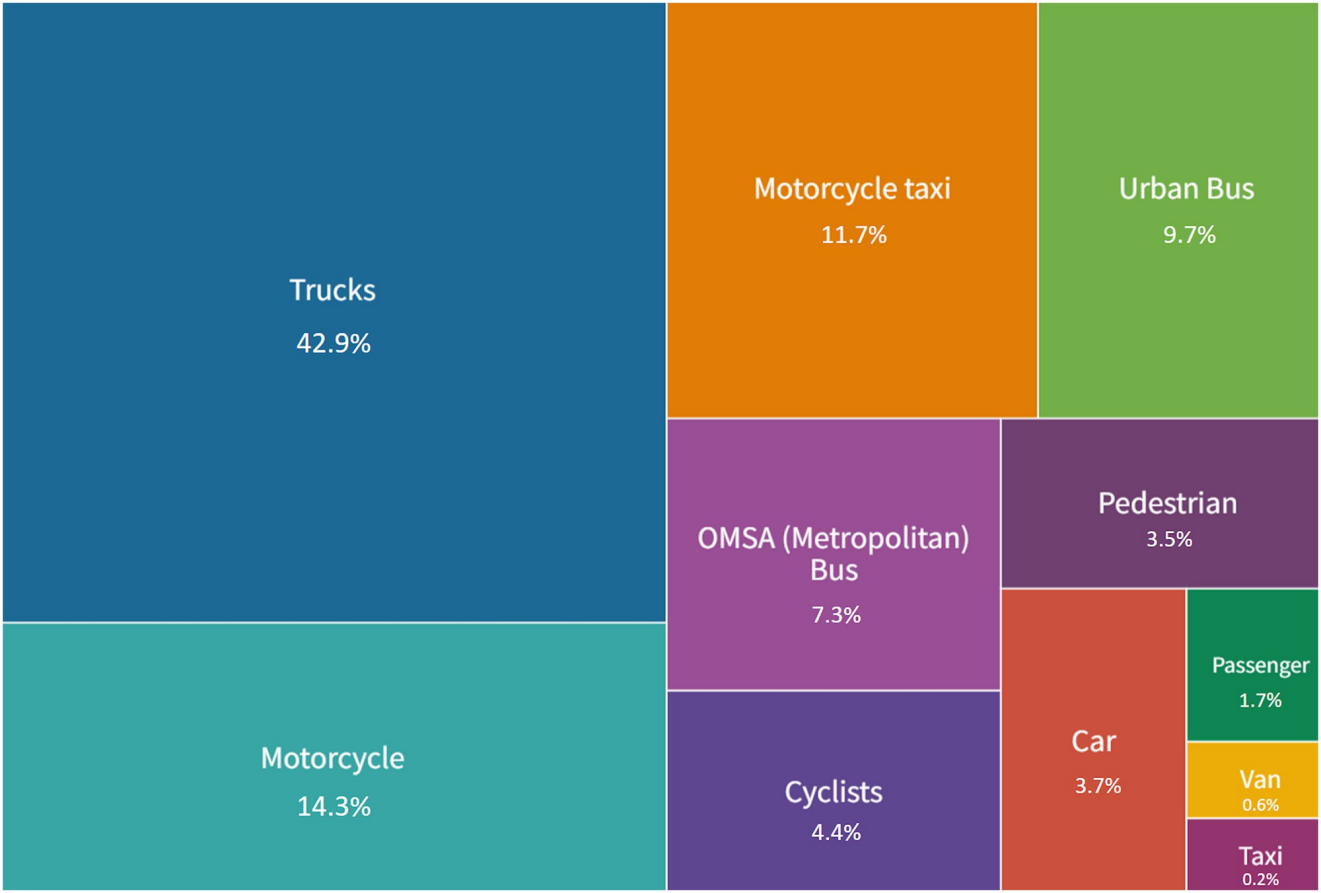
Perception of Dominicans on road users responsible for road crashes.

Regarding the type of user ‘causing crashes’, most participants agree that drivers have greater responsibility in road crashes (Fig 3). 42.9% believe professional truck drivers are responsible for most crashes. Meanwhile, a third of the sample points out to drivers of motorcycles (14.3% of private motorcycle drivers and 11.7% of motorcycle taxi drivers.

### Differences in the assessment of crash risk factors

Participants assessed the importance of different risk factors for road crashes in the Dominican Republic. The highest-scored crash contributor was reckless driving and/or traffic violations performed by drivers (*M* = 8.25; *SD* = 2.48). This is followed by the lack of laws and police supervision (*M* = 7 .19; *SD* = 3.12), poor condition of the vehicle (*M* = 7.18; *SD* = 3.03), and poor condition of the infrastructures (*M* = 7.06; *SD* = 3.19). The element perceived as the less relevant for the occurrence of crashes is reckless driving and infractions committed by pedestrians (*M* = 6.76; *SD* = 3.11).

**Fig 4:**
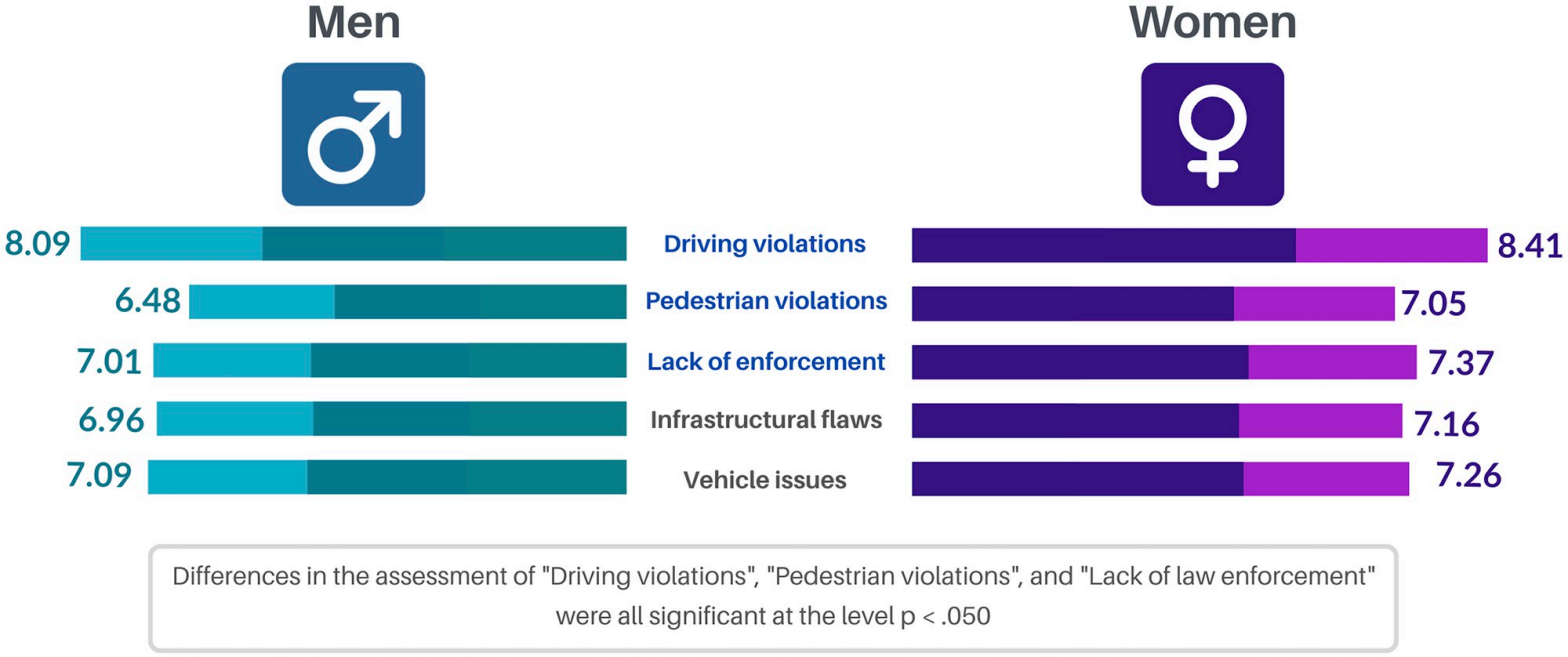
Assessment of the importance of risk factors for road crashes by sex of the respondent.

Comparative analyses (ANOVAs) have shown that there are no meaningful differences according to individuals’ habitat, driving licensing status, or regular driving. However, there have been statistically relevant differences regarding sex. In all cases, women are the ones depicted as the most important risk factors. There were three factors presenting significant sex-based differences:

- Driving violations (*F*_(1,1258)_ = 5.147; *p* = .023), in which men scored lower (*M* = 8.09; *SD* = 2.58) than women (*M* = 8.41; *SD* = 2.37).
- Pedestrian violations (*F*_(1,1258)_ = 10.782; *p* = .001), where men’s scores (*M* = 6.48; *SD* = 3.21) also tend to be lower than those reported by women (*M* = 7.05, *SD* = 2.97),
- Lack of law enforcement and police supervision (*F*_(1,1258)_ = 4.072; *p* = .044), in which women keep scoring higher (*M* = 7.37, *SD* = 3.19) than men (*M* = 7.01, *SD* = 3.27).
- On the other hand, the assessment of neither infrastructural flaws nor vehicle issues reported significant differences between men and women. Fig 4 graphically illustrates the mean scores reported by the two groups analyzed in the above mentioned five cases.

### Multiple linear regression

Finally, with the aim of assessing the effect of participants’ demographic features and perceptions about the five crash risk factors addressed in the questionnaire (i.e., driving and pedestrian violations, vehicle and infrastructure issues, and lack of enforcement) on the relevance they attribute to traffic crashes, a multiple linear regression model was built.

After controlling for age, educational level, and licensing status (dummy variable), a significant regression equation was found, with F_(8,1251)_ = 17.256, p< .001, and adjusted R^2^ = .132. The model’s constant was .519 + .106 (education) + .200 (driving violations) + .072 (pedestrian violations) + .113 (lack of law enforcement).

Namely, the model outcomes suggest that greater values on the relevance given to driving violations, pedestrian violations, and lack of law enforcement were the three significant predictors of attributing greater importance to traffic crashes occurring in the country. Among demographic factors, the only significant predictor was education, which also had a positive effect on the dependent variable. The full set of significant and non-significant variables included in the model, their coefficients and cut-off values are shown in Table 4.

**Table 4:** Linear regression model. Dependent variable: Importance attributed to traffic crashes in the Dominican Republic.

Predictor	Unstandardized Coefficients		Standardized Coefficients	t^(4)^	Sig.^(5)^	Adj. R^2(6)^	R^2(7)^
	B^(1)^	S.E.^(2)^	β ^(3)^				
(Constant)	.519	.041		12.539	< .001	.132	.139
Age	.000	.000	-.048	-1.455	.146		
Education	.021	.007	.106	3.175	.002		
Licensing status*	-.010	.011	-.026	-.856	.392		
Driving violations^8^	.203	.036	.200	5.689	< .001		
Pedestrian violations^8^	.056	.027	.072	2.068	.039		
Vehicle issues^8^	.039	.026	.050	1.461	.144		
Infrastructure issues^8^	.018	.025	.024	.702	.483		
Lack of law enforcement^8^	.086	.025	.113	3.362	< .001		

*Notes*: B^(1)^ = Unstandardized effect coefficient; S.E.^(2)^ = Standard Error; β ^(3)^ = Standardized effect coefficient (Beta–can be interpreted as controlling for effects of other variables); t^(4)^ = Value of the Student’s *t*-test; Sig.^(5)^ = *p*-value of the test; Adj. R2^(6)^ = Adjusted R-square; R^2(7)^ = Changes in R-square; ^(8)^ = The continuous variable has been logarithmically transformed for regression analysis;

* = Dummy variable.

Reference value = Licensed Driver.

## Discussion

The core aim of this study was to assess how Dominicans perceive some key road risky-related issues and to discuss them in the light of objective data. This aim has been raised on the basis of recent studies supporting the idea that an adequate balance between subjective evaluations and objective data would be a beneficial first step to promoting road safety among road users [54, 55]. However, as aforementioned, this issue remains, so far, understudied in the Dominican context. For this reason, the results of this study might help to establish a *baseline* on road safety-related perceptions and beliefs in the country.

The first issue addressed by this study was whether traffic crashes are perceived as an important issue by all Dominicans. In this regard, the descriptive results allow us to consider that a reasonable answer to this question could be “yes, but…”, as remarked by a recent study of 2015 conceptually analyzing different everyday problems of the country, in which traffic crashes did not even constitute a relevant matter to consider for most Dominicans [56]. In addition, other studies have suggested that this *slight* perceptual relevance of road safety matters could be strongly influenced by the lack of importance given to safety matters in different scenarios (e.g., mass media, social networks, and policymaking) [57].

Also, it is worth addressing the existence of gender differences regarding the importance attributed to traffic crashes in the country. Overall, the matter is consistently valued to a greater extent among women. Similar gender gaps in road safety-related issues have been found in previous studies dealing with cycling [41] and pedestrian [58] safety behaviors in Latin American countries. Further, Women use public transport frequently, whereas men drive motorized vehicles and are more involved in road crashes [59]. On the contrary, women are the ones who value the importance of road crashes greatly. This phenomenon is in line with previous research, where women tend to self-report higher levels of concern and risk perception about safety-related issues than men [60, 61].

A second issue addressed by this study was the coherence between individuals’ perceptions of the frequency of traffic crashes and the fatalities they carry with them. Overall, a considerable disagreement was found between what Dominicans perceived and real numbers. In broad terms, some interesting incongruities are worth mentioning. For instance, while most Dominicans state that it is important to keep working on preventing crashes and promoting road safety, their crash-related attributions point, on many occasions, to fate and luck [62–66].

Thirdly, and despite the lack of awareness and knowledge about the prevalence of crash rates, the perception of the typology of crashes is adjusted to the objective numbers registered by traffic institutions in the country. The majority of respondents agree that this issue’s most affected age group was *young people*, between 15 and 25 years (88.3%). This age group accounts for more than 30% of the deaths caused by this factor and, in fact, is objectively the most affected one. However, some underestimation may exist in the adult group between 40 and 60 years old and more than 60 years old, representing 21% and 15%, respectively.

Regarding official data, official records of the Dominican Republic indicate that 71.4% of deceased in road traffic crashes were motorcycle riders. Hierarchically speaking, they are followed by 12.5% pedestrians and 6.4% automobile rivers [47]. This is coherent with objective figures, showing that motorcycles were the type of vehicle most implicated in road crashes, representing about 68% of cases during the last few years [67]. These data can be considered very close to citizens’ perception, in which more than 80% believe the most affected users are private motorcycles and motorcycle taxi drivers. In addition to accounting for relatively realistic beliefs in this matter, this figure suggests a high awareness of the vulnerability of motorcycle riders, whose state of affairs remains a critical issue for road safety agencies in the Caribbean [68].

As for temporal crash features, the perception that there are more crashes during the weekends fits the objective data. In this case, 39.7% of crashes occur on Saturdays and Sundays, whereas 60.3% happen during the weekend. Hence, proportionally there are more significant crash rates on holidays. This is connected to leisure-related journeys [69]. Regarding the time of the day, day hours (from 8 a.m. to 8 p.m.) registered 64.8% of crashes, whereas 35.2% happened at night. In this case, citizen perception, albeit divided, considered there were more crashes at night.

Finally, and as for the effect of the five risk-related issues addressed in the study (i.e., driving and pedestrian violations, vehicle and infrastructure issues, and lack of enforcement) over the relevance individuals attribute to traffic crashes, two outcomes stand out: first, that the only demographic covariable significantly explaining the outcome was the educational level, while age and licensing status seem not to impact this perception. Second, that traffic violations constitute the strongest significant predictor, alongside lack of enforcement. Namely, both variables refer (although in different ways) to, respectively, deliberate road risk behaviors, and law-related actions aimed at reducing them [70]. Although the model is limited in terms of the number of variables and spheres addressed, these findings support the assumption that the behavioral domain might remain a key matter to be addressed in the region, both at the perceptual and the observational level [71].

### Limitations of the study and further research

Although this nationwide research had some key strengths, such as (*i*) addressing a scarcely studied issue in the region and (*ii*) privileging parsimonious (and literature-based) interpretations, it is worth acknowledging some shortcomings potentially affecting the study outcomes, as well as their validity.

Firstly, regardless of the anonymous approach to participants, we cannot assure the non-interference of common method biases (branded in the literature as CMBs). This study addressed noticeably sensitive topics (e.g. personal beliefs and perceptions) which can be affected by phenomena such as social desirability and politically correct answers. Therefore, readers are invited to interpret this data with caution, as we are unable to guess the actual influence of these biases in this type of design.

Also, it is worth remarking that the purpose of the linear regression model was to assess the effect of participants’ demographic features and perceptions about five crash risk factors on the relevance they attribute to traffic crashes. The model scope is thus limited to the data provided regarding a certain number of variables, thus constraining its capacity to explain the dependent variable multi-dimensionally. Said otherwise, there might be several other factors not addressed in this research potentially influencing citizens’ beliefs and perceptions. Further, even though it practically affects all current empirical research, the latest social changes in terms of transportation and concerns raised by the recent COVID pandemic should be considered for time-framing the results presented in this research.

Lastly, we would like to encourage researchers studying this topic to carry out complementary data collection techniques (e.g., surveys, focus groups), in order to increase the understanding of the topic “beyond numbers”.

## Conclusions

This research constitutes the first approach to road safety and crash-related perceptions and beliefs in the Dominican Republic. Overall, the results of this study suggest that:

- Although there is a relatively high valuation of traffic crashes as an important problem for the country, this perception is stronger among Dominican women.
- Despite a relative awareness of their actual traffic crash features, Dominicans systematically underestimate the causes, frequency, and consequences of these crashes, including yearly fatality rates.
- Finally, among the five risk-related factors addressed in the study, perceptions of traffic violations and lack of law enforcement are significant predictors of the degree of relevance attributed to traffic crashes among Dominican road users.

The results of this study can have theoretical and practical implications for the Government authorities of the Dominican Republic and other emerging countries to design and develop specific preventative measures that align with society’s level of awareness and beliefs. In any case, it is necessary to continue to carry out regular assessments, especially in emerging countries, to determine the level of knowledge of their citizens about the prevalence of this problem, the associated risk factors, and the influence of the human factor in preventing road crashes.

## Supporting information

S1 File (ZIP)
